# Maximum inferior vena cava diameter predicts post-induction hypotension in hypertensive patients undergoing non-cardiac surgery under general anesthesia: A prospective cohort study

**DOI:** 10.3389/fcvm.2022.958259

**Published:** 2022-10-04

**Authors:** Hanying Zhang, Hongguang Gao, Yuanjun Xiang, Junxiang Li

**Affiliations:** Department of Anesthesiology, Third Affiliated Hospital of Chengdu Medical College and Pidu District People’s Hospital Chengdu, Chengdu, Sichuan, China

**Keywords:** general anesthesia, hypertension, inferior vena cava, post-induction hypotension, ultrasound

## Abstract

**Background:**

Inferior vena cava (IVC) ultrasonography is a reliable variable that predicts post-induction hypotension (PIH) in patients undergoing surgery under general anesthesia. However, in patients with hypertension, the predictive performance of ultrasound IVC measurements needs further exploration.

**Methods:**

This is a prospective cohort study. Adult patients with existing hypertension scheduled to undergo non-cardiac surgery under general anesthesia were eligible. An abdominal ultrasound examination was conducted immediately prior to anesthesia induction (0.03 mg kg^–1^ midazolam, 0.3 mg kg^–1^ etomidate, 0.4 μg kg^–1^ sufentanil, and 0.6 mg kg^–1^ rocuronium). IVC collapsibility index (IVC-CI) was calculated as (dIVC_max_–dIVC_min_)/dIVC_max_, where dIVC_max_ and dIVC_min_ represent the maximum and minimum IVC diameters at the end of expiration and inspiration, respectively. PIH was defined as a reduction of mean arterial pressure (MAP) by >30% of the baseline or to <60 mmHg within 10 min after endotracheal intubation. The diagnostic performance of IVC-CI, dIVC_max_, and dIVC_min_ in predicting PIH was also examined in a group of normotensive patients receiving non-cardiac surgery under the same anesthesia protocol.

**Results:**

A total of 51 hypertensive patients (61 ± 13 years of age, 31 women) and 52 normotensive patients (42 ± 13 years of age, 35 women) were included in the final analysis. PIH occurred in 33 (64.7%) hypertensive patients and 19 (36.5%) normotensive patients. In normotensive patients, the area under the receiver operating curve (AUC) in predicting PIH was 0.896 (95% confidence interval [CI]: 0.804–0.987) for IVC-CI, 0.770 (95% CI: 0.633–0.908) for dIVC_max_, and 0.868 (95% CI: 0.773–0.963) for dIVC_min_. In hypertensive patients, the AUC in predicting PIH was 0.523 (95% CI: 0.354–0.691) for IVC-CI, 0.752 (95% CI: 0.621–0.883) for dIVC_max_, and 0.715 (95% CI: 0.571–0.858) for dIVC_min_. At the optimal cutoff (1.24 cm), dIVC_max_ had 54.5% (18/33) sensitivity and 94.4% (17/18) specificity.

**Conclusion:**

In hypertensive patients, IVC-CI is unsuitable for predicting PIH, and dIVC_max_ is an alternative measure with promising performance.

**Clinical trial registration:**

[http://www.chictr.org.cn/], identifier [ChiCTR2000034853].

## Introduction

Preoperative volume deficiency is a major risk factor for post-induction hypotension (PIH) in patients undergoing surgery under general anesthesia (1). The risk of PIH is particularly high in patients with underlying chronic wasting disease and patients not managed with robust Enhanced Recovery After Surgery programs due to prolonged restriction of fluid intake (2). Ultrasound-derived parameters of the inferior vena cava (IVC) have been used to predict PIH (3). The best performing variable among these parameters is the IVC collapsibility index (IVC-CI, calculated as (dIVC_max_–dIVC_min_)/dIVC_max_, where dIVC_max_ and dIVC_min_ represent the maximum and minimum IVC diameter at the end-expiration and inspiration, respectively) (4, 5).

The predictive performance of ultrasound measurements of IVC (including IVC-CI and dIVC_max_) is compromised in patients undergoing vascular surgery (6). This is particularly problematic since patients with hypertension are more likely to develop PIH than normotensive patients. The incidence of PIH has been estimated to be as high as 65% in hypertensive patients versus 54.7% in normotensive patients (7). Accurate prediction of PIH is particularly meaningful since hypertensive patients have an increased risk of organ damage upon hypotension (8, 9). We, therefore, conducted a prospective cohort study to examine whether the IVC’s ultrasound-derived parameters, including IVC-CI, dIVC_max_ and dIVC_min_, could predict PIH in patients with comorbid hypertension.

## Materials and methods

### Patient population

This study was approved by the Medical Ethics Committee of Pidu District People’s Hospital (ID: [2020] #0169, on July 11, 2020). The study protocol was registered at the Chinese Clinical Trial Registry (registration number ChiCTR2000034853; date of registration, July 22, 2020). Written informed consent was obtained from all patients or their legal surrogates.

Adult hypertensive patients scheduled for elective non-cardiac surgery under general anesthesia with a single lumen endotracheal intubation were eligible. The diagnosis was based on elevated systolic blood pressure (SBP) > 140 mmHg and/or elevated diastolic blood pressure (DBP) > 90 mmHg upon at least three measurements in resting conditions (10) or regular treatment with antihypertensive agents. A separate group of adult normotensive patients scheduled for elective non-cardiac surgery under general anesthesia was recruited as a reference group.

Subjects with one or more of the following conditions were excluded: (1) American Society of Anesthesiologists (ASA) physical status IV or higher; (2) ascites; (3) implanted pacemaker/cardioverter; (4) secondary hypertension, and (5) predicted difficult airway. Subjects with one or more conditions were excluded from the final analysis: (1) patients with reintubation or prolonged intubation [defined as intubation time greater than 30 s (11)]; (2) insufficient ultrasound image quality; (3) anaphylactic shock; (4) blood pressure > 180/110 mmHg on at least two adjacent non-invasive monitoring separated by 1 min after intubation; (5) severe arrhythmia that affected circulatory stability after induction; and (6) inconsistent dIVC_max_ and dIVC_min_ at the baseline (i.e., >0.2 cm difference between any two of the three respiratory cycles).

### Inferior vena cava ultrasonography

**FIGURE 1:**
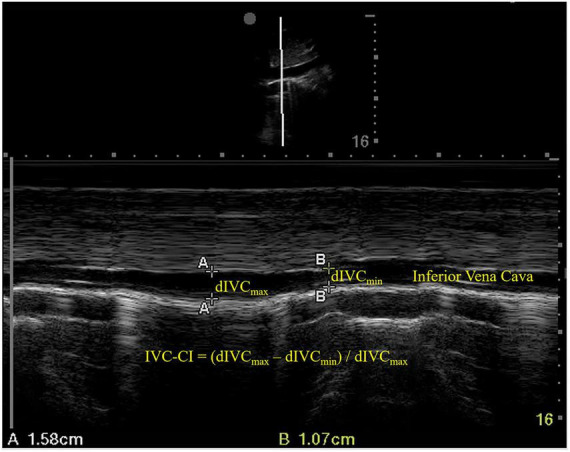
Ultrasound measurements of the inferior vena cava. dIVC_max_, maximum inferior vena cava diameter; dIVC_min_, minimum inferior vena cava diameter; IVC-CI, collapsibility index of inferior vena cava.

An ultrasound examination of the IVC was conducted immediately before anesthesia induction using a phased array probe (Sonosite Edge II) in M mode, with the patients in a supine position. The sampling site was placed 3 cm from the right atrium (Figure 1). dIVC_max_ (at the end of normal expiration) and dIVC_min_ (at the end of normal inspiration) were measured in three consecutive unforced respiratory cycles, and the results were averaged. IVC-CI was calculated as (dIVC_max_–dIVC_min_)/dIVC_max_ and expressed as a percentage (Figure 1).

### Anesthesia

Angiotensin receptor blockers (ARBs) and angiotensin-converting enzyme inhibitors (ACEIs) were discontinued on the day of surgery according to the recommendation by the American College of Cardiology/American Heart Association on Clinical Practice Guidelines (12). Calcium channel blockers, beta-blockers, and diuretics were continued.

Patients fasted starting from midnight before the day of surgery. Patients scheduled to undergo surgery in the afternoon received 10 ml kg^−1^ Ringer’s solution in the morning. An intravenous line was established (Ringer’s solution at 5 ml kg^–1^ h^–1^), and patients were sedated with midazolam (0.01 mg kg^–1^ intravenously) in the preparation room to relieve their anxiety. Upon entering the operating room, patients were monitored with an electrocardiogram (ECG), oxygen saturation (SpO_2_), and end-tidal carbon dioxide (EtCO_2_). The pre-induction mean arterial pressure (MAP) was used as the baseline MAP. After ultrasound examination of the IVC, anesthesia was induced with 0.03 mg kg^–1^ midazolam, 0.3 mg kg^–1^ etomidate, 0.4 μg kg^–1^ sufentanil, and 0.6 mg kg^–1^ rocuronium. Patients were intubated with a single-lumen endotracheal tube of appropriate size. Ringer’s solution was infused with 10 ml kg^−1^ h^−1^ throughout induction. Anesthesia was maintained with 1 vol% sevoflurane for 10 min after endotracheal intubation. Then anesthesia was maintained with propofol, sevoflurane, or both combined with remifentanil at bispectral index (BIS) 40–60 until the surgery finished. Blood pressure was monitored non-invasively at the 1-minute interval for 10 min (13). Upon completion of the surgery, all the anesthetics were discontinued, and the neuromuscular block was reversed with 0.02 mg kg^–1^ neostigmine and 0.01 mg kg^–1^ atropine. When the patient was fully awake, the endotracheal tube was removed, and all patients were transferred to the post-anesthesia care unit (PACU).

### Outcomes

The primary outcome was PIH, defined as the reduction of MAP within 10 min after endotracheal intubation to either <60 mmHg or to >30% of the pre-induction baseline (13, 14). Intravenous ephedrine (3 mg) was used if MAP decreased to <55 mmHg or to >35% of the baseline. Atropine (0.3 mg) was used if HR decreased to <50 beats min^–1^.

### Statistical analysis

In a pilot study in 20 hypertensive patients, PIH occurred in nine (45%) of them. At the optimal cutoff, IVC-CI had 77.8% (7/9) sensitivity and 45.5 (5/11) specificity in predicting PIH; the area under the receiver operating characteristic (ROC) curve was 0.535. At the optimal cutoff, dIVC_max_ had 44.4% (4/9) sensitivity and 100% (11/11) specificity in predicting PIH; the area under the ROC curve (AUC) was 0.732. To achieve a reasonable estimate of sensitivity and specificity, we set the sample size at 60. A group of 60 normotensive patients was also recruited.

For comparison between the patients with and without PIH, continuous variables were analyzed using Student’s *t*-test for independent samples and expressed as mean ± standard deviation if normally distributed (as assessed using the Shapiro–Wilk test) or analyzed using the Mann–Whitney U-test and expressed as median (interquartile range) otherwise. Categorical variables were analyzed using the χ*^2^* test and expressed as numbers (percentage). Diagnostic performance was examined using specificity, sensitivity, and AUC under the ROC curves. *P* < 0.05 was considered statistically significant. IBM SPSS Statistics for Windows, Version 26.0 (IBM Corp, USA) was used for statistical analyses.

## Results

### Patient characteristics

**FIGURE 2:**
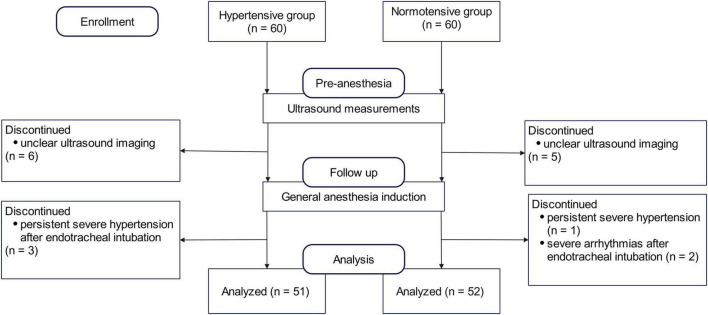
Flowchart of the hypertensive and normotensive group.

A total of 60 hypertensive patients were enrolled. The final analysis included 51 patients (61 ± 13 years of age; 31 women). A separate group of 60 normotensive patients was enrolled as a reference; the final analysis included 52 patients (42 ± 13 years of age, 35 women) in the normotensive group (Figure 2). Demographic and baseline characteristics are shown in Table 1. The antihypertensive agents in hypertensive patients included calcium channel blockers (22, 43.1%), angiotensin receptor blockers (ARBs) (9, 17.6%), beta-blockers (2, 3.9%), and diuretics (1, 2%). Compared to the normotensive group, patients in the hypertensive group were older and had higher ASA grades.

**TABLE 1:** Demographic and baseline characteristics of the hypertensive versus normotensive patients.

	Hypertensive patients (*n* = 51)	Normotensive patients (*n* = 52)
Age (year)	61 ± 13	42 ± 13
Male sex, *n* (%)	20 (39.2)	17 (32.7)
BMI (kg m^–2^)	24.2 (22.8, 26.2)	23 ± 3.1
Charlson comorbidity index	2 (1, 3)	0 (0, 2)
**ASA status, *n* (%)**		
I	0 (0)	13 (25)
II	32 (62.7)	39 (75)
III	19 (37.3)	0 (0)
**Type of surgery, *n* (%)**		
Gynecological	16 (31.4)	15 (28.8)
General	26 (51.0)	21 (40.4)
Orthopedic	3 (5.9)	11 (21.2)
Urological	6 (11.8)	5 (9.6)

Data were presented as Mean ± SD, number (%), or median (range). BMI, body mass index; ASA, American Society of Anesthesiologists. The data of BMI in hypertensive patients and Charlson comorbidity index in both groups were non-normally distributed after the Shapiro–Wilk normality test.

### Post-induction hypotension in normotensive patients

**FIGURE 3:**
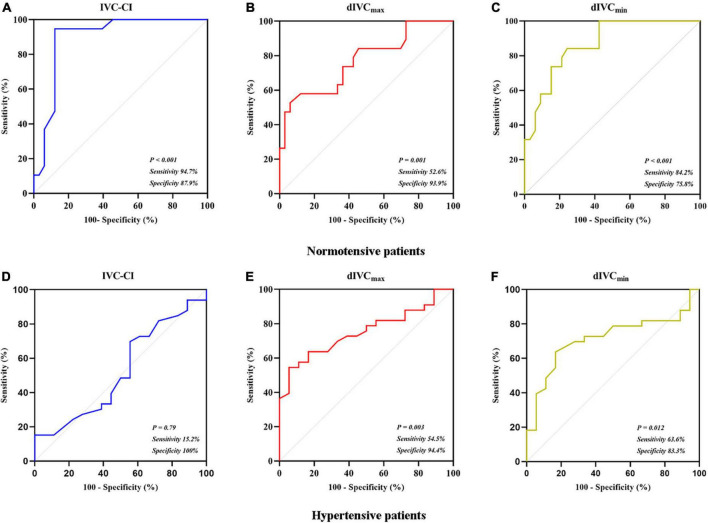
Receiver operating characteristic curves. **(A)** IVC-CI in predicting PIH in normotensive patients; **(B)** dIVC_max_ in predicting PIH in normotensive patients; **(C)** dIVC_min_ in predicting PIH in normotensive patients; **(D)** IVC-CI in predicting PIH in hypertensive patients; **(E)** dIVC_max_ in predicting PIH in hypertensive patients; and **(F)** dIVC_min_ in predicting PIH in hypertensive patients.

In the 52 normotensive patients, PIH occurred in 19 (36.5%) patients in the final analysis. In comparison to the patients who did not develop PIH, patients with PIH had higher IVC-CI (*P* < 0.001), smaller dIVC_min_ (*P* < 0.001) and dIVC_max_ (*P* = 0.001) (Table 2). At the optimal cutoff (43%), IVC-CI had a 0.896 AUC under the ROC curve (95% confidence interval [CI]: 0.804–0.987). The sensitivity and specificity were 94.7% (18/19) and 87.9% (29/33), respectively (Figure 3A). At the optimal cutoff (1.29 cm), dIVC_max_ had a 0.770 AUC under the ROC curve (95% CI: 0.633–0.908). The sensitivity and specificity were 52.6% (10/19) and 93.9% (31/33), respectively (Figure 3B). At the optimal cutoff (0.88 cm), dIVC_min_ had 0.868 AUC under the ROC curve (95% CI: 0.773–0.963). The sensitivity and specificity were 84.2% (16/19) and 75.8% (25/33), respectively (Figure 3C).

**TABLE 2:** Baseline characteristics and ultrasound-based inferior vena cava (IVC) parameters in patients with versus without post-induction hypotension (PIH).

	Hypertensive patients		*P*-value	Normotensive patients		*P*-value
			
	No PIH (*n* = 18)	PIH (*n* = 33)		No PIH (*n* = 33)	PIH (*n* = 19)	
Age (year)	58 ± 12	63 ± 13	0.156	40 ± 12	46 ± 12	0.074
Male sex, *n* (%)	8 (44.4)	12 (36.4)	0.572	14 (42.4)	3 (15.8)	0.049
BMI (kg m^–2^)	25.1 (23, 26.2)	24 (22.4, 26)	0.442	22.9 ± 3.2	23.2 ± 3.1	0.81
Charlson comorbidity index	2 (1, 3)	2 (1, 3)	0.421	0 (0, 1.5)	0 (0, 2)	0.851
ASA status, *n* (%)			0.101			0.868
I	0 (0)	0 (0)		8 (24.2)	5 (26.3)	
II	14 (77.8)	18 (54.5)		25 (75.8)	14 (73.7)	
III	4 (22.2)	15 (45.5)		0 (0)	0 (0)	
Baseline MAP (mmHg)	108 ± 9	109 ± 9	0.886	89 ± 7	93 ± 9	0.08
IVC-CI (%)	38 (29, 45)	35 (30, 45)	0.79	33 ± 10	47 ± 4	<0.001
dIVC_max_ (cm)	1.56 (1.44, 1.81)	1.2 (1.05, 1.54)	0.003	1.68 ± 0.33	1.33 ± 0.34	0.001
dIVC_min_ (cm)	1.02 (0.92, 1.08)	0.78 (0.63, 1.01)	0.012	1.15 ± 0.33	0.71 ± 0.2	<0.001

Data were presented as Mean ± SD, number (%), or median (range). PIH, post-induction hypotension; BMI, body mass index; ASA, American Society of Anesthesiologists; dIVC_max_, maximum inferior vena cava diameter; dIVC_min_, minimum inferior vena cava diameter; IVC-CI, collapsibility index of inferior vena cava. The data of BMI, Charlson comorbidity index, IVC-CI, dIVC_max_, and dIVC_min_ in hypertensive patients were non-normally distributed after the Shapiro-Wilk normality test. The data of the Charlson comorbidity index in normotensive patients were non-normally distributed after the Shapiro–Wilk normality test.

### Post-induction hypotension in hypertensive patients

In the 51 hypertensive patients, in the final analysis, PIH occurred in 33 (64.7%) patients. In comparison to the patients who did not develop PIH, patients with PIH had smaller dIVC_max_ (*P* = 0.003) and dIVC_min_ (*P* = 0.012) (Table 2). At the optimal cutoff (50%), IVC-CI had a 0.523 AUC under the ROC curve (95% CI: 0.354–0.691). The sensitivity and specificity were 15.2% (5/33) and 100% (18/18), respectively (Figure 3D). At the optimal cutoff (1.24 cm), dIVC_max_ had 0.752 AUC under the ROC curve (95% CI: 0.62–0.883). The sensitivity and specificity were 54.5% (18/33) and 94.4% (17/18), respectively (Figure 3E). At the optimal cutoff (0.88 cm), dIVC_min_ had 0.715 AUC under the ROC curve (95% CI: 0.571–0.858). The sensitivity and specificity were 63.6% (21/33) and 83.3% (15/18), respectively (Figure 3F).

## Discussion

The current study confirmed a higher rate of PIH in hypertensive patients undergoing non-cardiac surgery under general anesthesia. Consistent with previous studies (3, 15), IVC-CI could predict PIH in the normotensive group. dIVC_max_ and dIVC_min_ also had good performances. In hypertensive patients, however, the predictive performance of IVC-CI was very poor. In contrast, dIVC_max_ predicted PIH with a reasonable performance. At the optimal cutoff (1.24 cm), dIVC_max_ had 54.5% sensitivity and 94.4% specificity. The predictive performance of dIVC_min_ was between IVC-CI and dIVC_max_.

The optimal IVC-CI cutoff in normotensive patients (43%) was consistent with a previous study by Purushothaman et al. (16), who selected propofol as the anesthesia inducer. The optimal dIVC_max_ cutoff in normotensive patients (1.29 cm) was comparable to previous studies (13). These results supported using ultrasound-based IVC parameters, including IVC-CI, dIVC_max_, and dIVC_min_, in predicting PIH in normotensive patients undergoing non-cardiac surgery under general anesthesia.

IVC-CI had very poor predictive performance in hypertensive patients in the current study. Such a finding is consistent with previous studies (6) and highlights the need for an alternative measure to predict PIH. The following reasons may explain this result. First, hypertensive patients have impaired automatic blood pressure regulation and are more likely to develop hypotension after general anesthesia induction (17). Second, hypertensive patients have much lower venous compliance than those with normal blood pressure (18). A decrease in capacity, a change in venous compliance, or both can affect the volume change in the inferior vena cava. As venous compliance decreases, volume change in the inferior vena cava decreases (19). Accordingly, the degree to which IVC-CI reflects the true volume status is lower than in patients with normal blood pressure.

In contrast to the very poor performance of IVC-CI, dIVC_max_ had reasonable performance in predicting PIH in the current study, with 0.752 AUC under the ROC curve. A recent study found better predictive performance with dIVC_max_ (than with IVC-CI) in elderly patients receiving gastroscopy under general anesthesia (20). Despite the encouraging findings, the sensitivity and specificity of dIVC_max_ in predicting PIH are far from the levels required for use in daily practice. In a previous study by Aissaoui et al. (21), ΔVTI-PLR (velocity time integral of the left ventricular outflow tract changes induced by passive leg raise) predicted PIH with a 0.89 AUC under the ROC curve (95% CI: 0.80–0.97) at a cutoff of 18% (88% sensitivity and 84% specificity) in patients >50 years of age. Similar to ultrasound-based IVC parameters (22), the mechanism of ΔVTI-PLR in predicting PIH is based on fluid responsiveness assessment (23). ΔVTI-PLR was not selected in the current study due to technical difficulty (24).

The prevalence of hypertension in the elderly population is much higher than in younger people (25). As such, the interaction of age and hypertension in PIH and the use of ultrasound-based IVC parameters in predicting PIH require further study.

Angiotensin receptor blockers(ARBs) and Angiotensin-Converting Enzyme Inhibitors(ACEIs) are the most commonly used antihypertensive drugs (26). The potential risks and benefits of ACE inhibitors in the perioperative setting are still controversial (12). In a previous study, patients who discontinued ACEIs/ARBs before surgery were less likely to suffer PIH than those continuing these medications (27). In the current study, ARBs/ACEIs were discontinued on the morning of surgery to minimize PIH. Whether dIVC_max_ could predict PIH in hypertensive patients not discontinuing ARBs/ACEIs requires further investigation.

There were several limitations to the current study. First, this is a single-center study with a relatively small sample size. As such, the results must be considered preliminary and require verification by future studies. Secondly, the patient population is heterogeneous regarding blood pressure control status: some patients were on treatment with antihypertensive drugs, and others were not. Third, we did not adopt a stringent Enhanced Recovery After Surgery (ERAS) program during the study period (28). All patients were fasted starting at midnight on the day before surgery. For patients undergoing surgery in the afternoon, fluid was given in the morning, as described earlier. Restricted food and water intake conceivably made the patients more susceptible to PIH. Whether the results from the current study apply to the more stringent ERAS program settings is unknown.

Last but not least, the performance of dIVC_max_ is not optimal. The 54% sensitivity at the 1.24 cm cutoff is clearly far below the criteria for use as a standard diagnostic test. But considering the limited options in predicting post-induction hypotension in hypertensive patients and the wide availability of ultrasound examinations, we believe dIVC_max_ is a useful measure to assess the risk of post-induction hypotension.

## Conclusion

IVC-CI could reliably predict PIH in normotensive patients undergoing non-cardiac surgery under general anesthesia but had very poor predictive performance in hypertensive patients. dIVC_max_ is an alternative measure that could predict PIH with some but limited accuracy.

## Data availability statement

The raw data supporting the conclusions of this article will be made available by the authors, without undue reservation.

## Ethics statement

The studies involving human participants were reviewed and approved by the Medical Ethics Committee of Pidu District People’s Hospital, Chengdu, China [reference (2020) No. 0169]. The patients/participants provided their written informed consent to participate in this study. Written informed consent was obtained from the individual(s) for the publication of any potentially identifiable images or data included in this article.

## Author contributions

JL, HZ, and HG designed and conducted the study. HG, HZ, and YX were responsible for the collection of data. HZ and JL analyzed and interpreted the data and drafted and revised the manuscript. All authors contributed to the article and approved and took accountability for the final manuscript.
